# Phytochemical Composition of the Fruit of Large Cranberry (*Vaccinium macrocarpon* Aiton) Cultivars Grown in the Collection of the National Botanic Garden of Latvia

**DOI:** 10.3390/plants12040771

**Published:** 2023-02-08

**Authors:** Rima Šedbarė, Ginta Jakštāne, Valdimaras Janulis

**Affiliations:** 1 Department of Pharmacognosy, Faculty of Pharmacy, Lithuanian University of Health Sciences, 50166 Kaunas, Lithuania; 2 Department of Food, Aromatic and Medicinal Plants, National Botanic Garden, 2169 Salaspils, Latvia

**Keywords:** proanthocyanidins, flavonols, anthocyanins, triterpenoids

## Abstract

In this study, we conducted a qualitative and quantitative analysis of anthocyanins, proanthocyanidins, flavonols and triterpenoids in samples of introduced and bred large cranberry fruits from the collection of the National Botanic Garden of Latvia. The highest total anthocyanin levels (8638–9316 µg/g) were detected in the fruit samples of the cranberry cultivars ‘Black Veil’, ‘Franclin’ and ‘Early Black’. The highest total proanthocyanidin levels (2775–3389 µg/g) were found in cranberries of cultivars ‘Kalnciema Agrā’, ‘Kalnciema Tumšā’, ‘Searles’, ‘Howes’, and ‘Kalnciema Ražīgā’. The highest levels of flavonol compounds (1373–1402 µg/g) were detected in cranberries of cultivars ‘Howes’, ‘Black Veil’ and ‘Salaspils Melnās’. The highest levels of triterpenoids (5292–5792 µg/g) were determined in cranberries of cultivars ‘Kalnciema Agrā’, ‘Septembra’, ‘Džbrūklene’ and ‘Early Black’. The results of our study allow for the estimation of differences in the content of secondary metabolites in the fruit samples of the studied cranberry cultivars and for the selection of promising cultivars for further introduction and cultivation in the climatic conditions of the Baltic countries. These results are also important for the selection of the most promising cranberry cultivars for the preparation of cranberry raw material, and the high-quality composition of plant material ensures the effectiveness of cranberry supplements and other cranberry preparations.

## 1. Introduction

Large cranberry (*Vaccinium macrocarpon* Aiton) is a perennial evergreen plant that grows naturally in North America [1]. Cranberry breeding and cultivation studies were initiated in the USA in the 19th century with cranberry plant taxa growing in natural cenopopulations [2]. From cultivars that were cultivated in the 20th century, the Big Four (‘Early Black’, ‘Howes’, ‘McFarlin’ and ‘Searles’) were selected [2]. Scientific work on cranberry breeding, hybridization and introduction is being carried out with the aim of developing cultivars with disease resistance and high yields [3]. Currently, the world’s largest industrial production of cranberry fruit comes from the cranberry cultivars ‘Stevens’, ‘Pilgrim’ and ‘Mullica Queen’ [2,3].

Under the climatic conditions of the Baltic region, the small cranberry (*Vaccinium oxycoccus* L.) is the predominant species in the natural cenopopulations of raised bogs and intermediate bogs [4,5]. In the Baltic region, cranberries are traditionally used in folk medicine and for food [6]. As the area of natural cranberry plantations has been reduced by anthropogenic activities, research and breeding for the introduction and cultivation of large cranberries has started [7].

In Latvia, important breeding and introduction studies on cranberries have been carried out at the National Botanic Garden of Latvia in Salaspils. The first *Vaccinium macrocarpon* cultivars were introduced into the National Botanic Garden of Latvia in 1972 from the Massachusetts Cranberry Research Station in the USA. Later, the collection of the Botanic Garden was supplemented with other cultivars bred in the USA. Some of the cranberry cultivars in the collection have been developed by researchers at the National Botanic Garden of Latvia in Salaspils. The breeder, doctor of biology Alfreds Ripa, has carried out research for breeding the cranberry cultivars ‘Kalnciema Agrā’ (1998), ‘Kalnciema Tumšā’ (1998), ‘Kalnciema Ražīgā’ (1998) and ‘Septembra’ (1998), as well as cranberry–lingonberry hybrid cultivars ‘Tīna’ (2006), ‘Dižbrūklene’ (1997), ‘Salaspils Agrās’ (1996) and ‘Salaspils Melnās’ (1996). He has also prepared recommendations for their cultivation in the climatic conditions in Latvia.

The production of cranberry fruit preparations in Latvia has been increasing over the last 20 years [6,8]. Industrial cranberry plantations have been expanded, and consumer interest in cranberry fruit and cranberry products has increased due to their health benefits [6,8]. European countries manufacture a small proportion of the world’s cranberry fruit production [9]; thus, research into the introduction of new cranberry cultivars, the phytochemical composition of their fruit and the selection of the most promising cranberry cultivars for growing in the Baltic countries remains relevant [10]. The main objectives of cranberry cultivar selection and cultivation are to select early-maturing cranberry cultivars with fruit of good marketable appearance, uniform size, intense red color, maximal amounts of bioactive compounds, high yields and disease resistance [11].

The content of bioactive compounds in cranberries is determined by climatic factors (temperature, light, soil chemistry and moisture) and the genetic characteristics of the plant [12]. Studies on the phytochemical composition and quantitative analysis of cranberry fruit can help to assess patterns of the accumulation of biologically active compounds in different cranberry cultivar fruits during ripening and to ensure the preparation of high-quality plant material [13].

Cranberries and their processed products are used in the confectionery industry (jams, syrups, marmalades, baked goods and yogurts) and in the production of wine and juice [14,15]. Cranberry raw material is used in the development and production of high-quality cranberry fruit pharmaceuticals, extracts and food supplements [16]. The use of pharmaceutical preparations and food supplements is important for health promotion and disease prevention, and therefore, products must be made from high-quality cranberry raw material of known composition. Studies on cranberry fruit cultivars enable the selection of the cranberry cultivars with the highest levels of bioactive compounds in the raw fruit material.

The purpose of this study was to estimate the qualitative and quantitative compositions of triterpenoids, proanthocyanidins, flavonols and anthocyanins in fruit samples of large cranberry cultivars grown in the collection of the National Botanic Garden of Latvia. The conducted study can provide new information on the variability in the phytochemical composition of cranberries and is significant for the identification of the most promising cranberry cultivars for growing in the climatic conditions of Latvia and other countries of the Baltic region. The climatic conditions in these countries are similar, and therefore, the results of our research are relevant for scientists in these countries conducting research on breeding and introducing cranberry cultivars [10].

## 2. Results and Discussion

### 2.1. Determination of the Quantitative Composition of Anthocyanins and Anthocyanidins in Fruit Samples of Large Cranberry Cultivars

In cranberry breeding and introduction, the bright red color of cranberry fruit is one of the important criteria for cultivar selection [2,11]. The red color of the fruit is determined by the content of the plant pigments’ anthocyanins and anthocyanidins [17]. Anthocyanins have been shown to have antioxidant [18], anti-inflammatory [19] and symbiotic gut-bacteria-enhancing [20] effects, among others.

**Figure 1:**
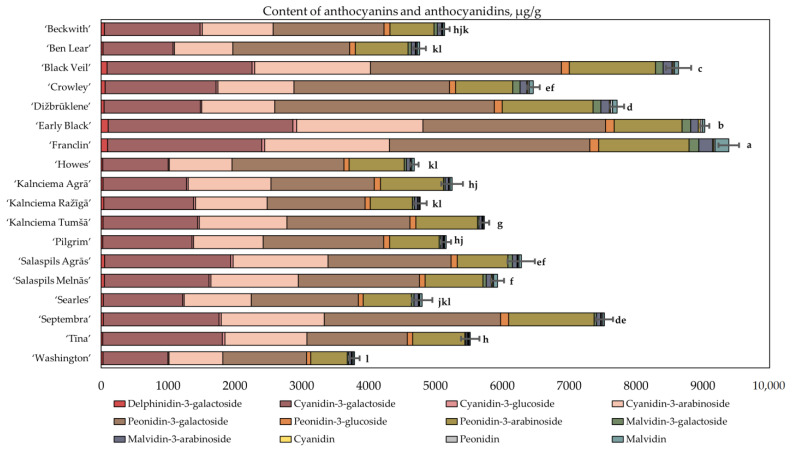
Variability in the amounts of anthocyanins and anthocyanidins in fruit samples of large cranberry cultivars. Statistically significant differences in total anthocyanin content between samples of cranberry cultivars are marked with different letters (*p* < 0.05).

We analyzed the compositions of anthocyanins and anthocyanidins in cranberries of the cultivars ‘Beckwith’, ‘Ben Lear’, ‘Black Veil’, ‘Crowley’, ‘Franclin’, ‘Kalnciema Agrā’, ‘Kalnciema Tumšā’, ‘Pilgrim’, ‘Salaspils Agrās’, ‘Salaspils Melnās’, ‘Searles’, ‘Septembra’, ‘Tīna’, ‘Dižbrūklene’, ‘Early Black’, ‘Howes’, ‘Kalnciema Ražīgā’ and ‘Washington’ (Figure 1). Four predominant major anthocyanins were found in the studied samples: cyanidin-3-galactoside (18.64–32.36%), cyanidin-3-arabinoside (14.18–23.63%), peonidin-3-galactoside (27.19–42.54%) and peonidin-3-arabinoside (11.26–17.91%). The other anthocyanins and anthocyanidin group compounds detected in the samples were lower in quantitative composition, including delphinidin-3-galactoside (0.39–1.13%), cyanidin-3-glucoside (0.27–0.76%), peonidin-3-glucoside (1.39–1.79%), malvidin-3-galactoside (0.25–1.73%), cyanidin (0.16–0.34%), malvidin-3-arabinoside (0.44–2.21%), peonidin (0.21–0.44%) and malvidin (0.27–2.21%).

Viskelis et al. conducted a study on the cranberry cultivars “Stevens”, “Pilgrim”, “Ben Lear” and “Black Veil” grown under Lithuanian climatic conditions and found that four major anthocyanins were predominant, and their quantitative compositions are consistent with the results of our study [5]. Česonienė et al. investigated the quantitative compositions of anthocyanins in the fruit juice of the cranberry cultivars ‘Howes’, ‘Black Veil’, ‘Washington’, ‘Stevens’, ‘Franklin’, ‘Early Richard’, ‘Searles’, ‘Pilgrim’ and ‘Le Munyon’ grown in Lithuania and found that four main anthocyanins are predominant in the juice, like the fruit samples [10]. The results of other researchers studies confirm the trends in the quantitative compositions of the four main anthocyanins observed in our studied fruit samples.

The total amounts of anthocyanins and anthocyanidins in the analyzed cranberries varied from 3788.33 ± 78 µg/g to 9316.32 ± 154 µg/g. The highest total anthocyanin content (9316.32 ± 154 µg/g) was determined in the cranberry cultivar ‘Franclin’ (Figure 1). The lowest total anthocyanin content (3788.33 ± 78 µg/g) was determined in fruit samples of the cranberry cultivar ‘Washington’, which was not statistically significantly different from the content determined in cranberries of the cultivars ‘Ben Lear’, ‘Howes’ and ‘Kalnciema Ražīgā’.

Brown et al. carried out a quantitative analysis of anthocyanin content in fruit samples of cultivars ‘Stevens’, ‘Pilgrims’, ‘GH 1’, ‘Bergman’ and ‘Ben Lear’. The total anthocyanin content varied from 2810 µg/g to 7980 µg/g [21]. Gardana et al. investigated the quantitative compositions of anthocyanins in the cranberries of cultivars ‘Bergman’, ‘Stevens’, ‘Howes’ and ‘Ben Lear’ and established that the total anthocyanin content varied from 2400 µg/g to 5600 µg/g [22].

Gardana et al. established that the levels of anthocyanins in the fruit samples of the ‘Howes’ cultivar were 5070 µg/g, which was higher than the amount (4683 µg/g) found in the fruit samples of this cultivar in our study. Gardana et al. found a cumulative variation in anthocyanin content from 2650 µg/g to 4140 µg/g in cranberries of the ‘Ben Lear’ cultivar. The total anthocyanidin content found by the researchers was lower than our finding of 4766 µg/g in the fruit samples of this cultivar [22].

Urbstaite et al. investigated samples of cranberry cultivars grown under Lithuanian climatic conditions and established that the total anthocyanin and anthocyanidin levels varied from 1950 µg/g to 8130 µg/g in the samples of the studied cultivars [23]. Samples of the cranberry cultivars ‘Early Black’ and ‘Searles’ had, respectively, 4.5 and 1.5 times lower total levels of anthocyanins than those of the samples of these cultivars grown in the collection of the National Botanic Garden of Latvia [23]. The anthocyanin content in fruit samples of the ‘Howes’ cultivar grown under Lithuanian climatic conditions was 1.5 times higher than that in fruit samples of this cultivar grown in Latvia, whereas the anthocyanin levels in cranberries of the ‘Crowley’ cultivar were the same [23].

**Figure 2:**
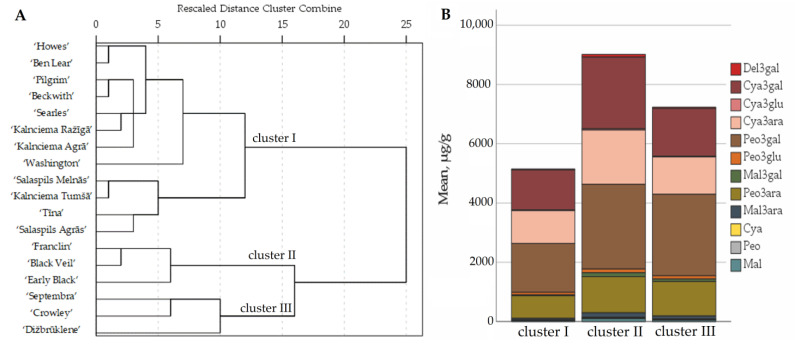
Dendrogram of distribution of cranberry cultivars into three groups according to anthocyanin content of the fruit samples (**A**). Graph of the distribution of the average anthocyanin content in the groups (**B**). Del3gal—delphinidin-3-galactoside; Cya3gal—cyanidin-3-galactoside; Cya3glu—cyanidin-3-glucoside; Cya3ara—cyanidin-3-arabinoside; Peo3gal—peonidin-3-galactoside; Peo3glu—peonidin-3-glucoside; Mal3gal—malvidin-3-galactoside; Peo3ara—peonidin-3-arabinoside; Mal3ara—malvidin-3-arabinoside; Cya—cyanidin; Peo—peonidin; Mal—malvidin.

The comparative analysis of the anthocyanins content in the fruit of large cranberry cultivars introduced and bred in the collection of the National Botanic Garden of Latvia was carried out by applying hierarchical cluster analysis. The cluster analysis of the fruit samples of the cultivars was carried out by analyzing the results of the qualitative and quantitative analysis of anthocyanins and anthocyanidins. Based on the quantitative composition of anthocyanins in cranberry fruit samples, the samples were divided into three clusters (Figure 2).

The cranberries of the cultivars ‘Beckwith’, ‘Howes’, ‘Kalnciema Agrā’, ‘Ben Lear’, ‘Kalnciema Ražīgā’, ‘Kalnciema Tumšā’, ‘Pilgrim’, ‘Salaspils Agrās’, ‘Salaspils Melnās’, ‘Searles’, ‘Tīna’ and ‘Washington’ were attributed to cluster I because their total anthocyanin content was lower than the average total anthocyanin content of the tested cultivars, ranging from 3788 µg/g to 6284 µg/g. The cultivars ‘Tīna’, ‘Salaspils Melnās’, ‘Salaspils Agrās’ and ‘Kalnciema Tumšā’, developed by Latvian scientists, were attributed to the subgroup of cluster I, as they had a similar anthocyanin composition. The cranberry cultivars ‘Tīna’, ‘Salaspils Melnās’ and ‘Salaspils Agrās’ are hybrids of lingonberry and cranberry. The predominant anthocyanins found in lingonberry fruit samples were cyanidin-3-galactoside (58%), cyanidin3-arabinoside (35%) and cyanidin-3-glucoside (7%) [24]. However, the anthocyanin profiles found in fruits of lingonberry–cranberry hybrid cultivars (cyanidin-3-galactoside (27%), cyanidin-3-arabinoside (20%), peonidin-3-galactoside (27%), and peonidin-3-arabinoside (15%)) were consistent with the profiles of anthocyanins found in fruits of other cranberry cultivars (Figure 1).

Cluster II consisted of the large cranberry cultivars ‘Black Veil’, ‘Franclin’ and ‘Early Black’, with anthocyanin levels ranging from 8638 µg/g to 9316 µg/g (Figure 1). The total anthocyanin content in the cranberry fruit samples was 1.5 times higher than the average total anthocyanin content of the tested cultivars. Sapers et al. classified the cranberry cultivars ‘Black Veil’, ‘Franclin’ and ‘Early Black’ as cultivars accumulating high anthocyanin levels [25]. The cranberry cultivar ‘Franclin’ is a hybrid obtained by combining the ‘Early Black’ and ‘Howes’ cultivars [2]. The total anthocyanin content of ‘Franclin’ fruit samples was not statistically different from the total anthocyanin content of ‘Early Black’ fruit samples and was two times higher than the anthocyanin content found in fruit samples of the ‘Howes’ cultivar. Diaz-Garcia et al. determined that cranberries of second- and third-generation cycle cultivars had a higher anthocyanin content than that of fruit samples of early-selection cultivars [3].

Cluster III included fruit samples of the cultivars ‘Septembra’, ‘Dižbrūklene’ and ‘Crowley’, where the total anthocyanin content was found to be 1.2 times higher than the average anthocyanin content of all the tested cultivars, ranging from 6465 µg/g to 7719 µg/g. Tikuma et al. found that ‘Septembra’, a cultivar developed in Latvia, has large, intensely red berries, and that the anthocyanin content of the fruit samples of this cultivar is higher than the anthocyanin content of the fruit samples of the cultivars ‘Stevens’, ‘Bergman’ and ‘Pilgrim’, and lower than the anthocyanin content of the fruit samples of the ‘Early Black’ cultivar [26]. Urbstaite et al. determined that the total anthocyanin content in cranberries of the hybrid ‘Crowley’ (a hybrid of ‘Prolific’ and ‘Mc Farlin’ cultivars) is higher than in cranberries of both ‘Mc Farlin’ and ‘Prolific’ cultivars [23].

The highest levels of anthocyanins were found in cranberry cultivars assigned to clusters II and III. The cultivation of ‘Black Veil’, ‘Franclin’, ‘Early Black’, ‘Septembra’, ‘Dižbrūklene’ and ‘Crowley’ cultivars, which were assigned to these clusters, is promising, as the plants accumulate higher amounts of anthocyanins in their fruit. The cultivation of these cranberry cultivars is relevant for the preparation of plant material with a high anthocyanin content. Anthocyanins determine the red color of the fruit, and the fruit of these cultivars may therefore have applications in the confectionery and bakery industry as a natural coloring agent [27]. Anthocyanins are biologically active compounds, and thus, the fruit of the above-mentioned cultivars can be used as plant material for the production of dietary supplements and preparations whose effects are determined by anthocyanins [28].

### 2.2. Determination of the Quantitative Composition of Proanthocyanidins in Fruit Samples of Large Cranberry Cultivars

Proanthocyanidins are an important group of biologically active compounds with antibacterial [29] and anti-inflammatory [30] effects found in cranberry fruit samples. Spectrophotometric assays are often used to determine the proanthocyanidin content of cranberry plant material and its preparations [31,32,33].

**Figure 3:**
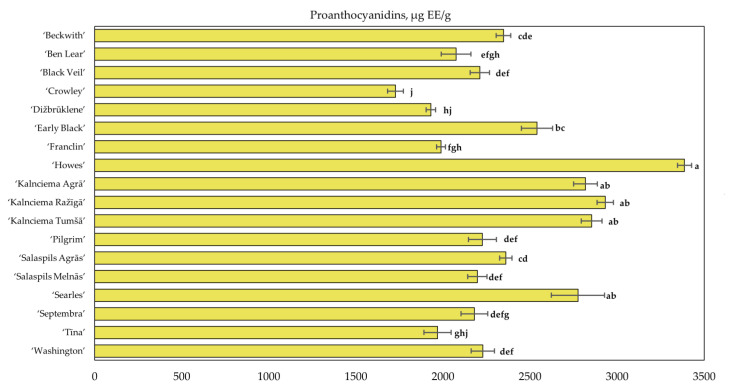
Variability in the total amount of proanthocyanidins in fruit samples of large cranberry cultivars. Statistically significant differences in proanthocyanidins content between samples of cranberry cultivars are marked with different letters (*p* < 0.05).

Using spectrophotometry, we determined that the total proanthocyanidin content in cranberries of cultivars grown in the collection of the National Botanic Garden of Latvia ranged from 1727.40 ± 45 µg EE/g to 3386.94 ± 41 µg EE/g (Figure 3). The mean total proanthocyanidin content of the cranberry fruit samples was calculated to be 2375.05 ± 63 µg EE/g. The highest proanthocyanidin content (3386.94 ± 41 µg EE/g) was determined in cranberries of the ‘Howes’ cultivar, whereas the lowest content (1727.40 ± 45 µg EE/g) was found in cranberries of the ‘Crowley’ cultivar. Šedbarė et al. analyzed fruit samples of the cranberry cultivars ‘Tina’, ‘Stevens’, ‘Pilgrim’, ‘Kalnciema agra’, ‘Bergman’, ‘Lemunyon’ and ‘Ben Lear’ grown in Latvia and determined that the total proanthocyanin content ranged from 3280 µg EE/g to 5990 µg EE/g [34]. Gu et al. analyzed cranberry fruit lyophilizate and found that the total proanthocyanidin content in the studied fruit samples was 4188 µg/g [35].

**Figure 4:**
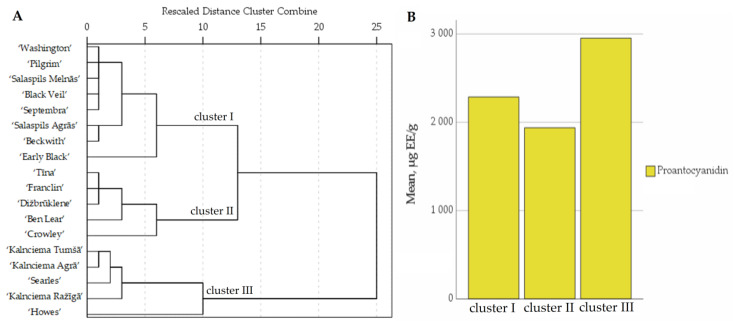
Dendrogram of distribution of cranberry cultivars into three groups according to proanthocyanidin content of the fruit samples (**A**). Graph of the distribution of the average proanthocyanidin content in groups (**B**).

The quantitative comparative analysis of the proanthocyanidin compositions of the fruit samples of cranberry cultivars grown in the National Botanic Garden of Latvia was carried out using hierarchical cluster analysis. The studied cranberry samples were divided into three groups (Figure 4).

The proanthocyanidin content of the fruit samples of cluster I cranberry cultivars ‘Washington’, ‘Septembra’, ‘Salaspils Agrās’, ‘Salaspils Melnās’, ‘Pilgrim’, ‘Early Black’, ‘Black Veil’ and ‘Beckwith’ was close to the average of the total proanthocyanidin content of all the tested cultivars. Cluster II consisted of the cranberry cultivars ‘Tīna’, ‘Franclin’, ‘Beech’, ‘Crowley’ and ‘Ben Lear’, in which the total proanthocyanidin content in the fruit samples was lower than the average total proanthocyanidin content of the samples of the tested cultivars.

The total proanthocyanidin content of the samples of the cranberry cultivars ‘Kalnciema Agrā’, ‘Kalnciema Tumšā’, ‘Searles’, ‘Howes’ and ‘Kalnciema Ražīgā’ assigned to cluster III was higher than the average proanthocyanidin content of the tested cultivars. The cranberry cultivars ‘Kalnciema Agrā’, ‘Kalnciema Tumšā’ and ‘Kalnciema Ražīgā’ developed in Latvia in 1998 were included in the cluster due to their high total proanthocyanidin content, which was not statistically significantly different from the average proanthocyanidin content of the tested cultivars. The high proanthocyanidin content found in the fruit samples of these cranberry cultivars bred in Latvia could be due to genetic factors. Vorsa et al. pointed out that the total proanthocyanidin content of cranberry fruit samples is influenced by environmental and genetic factors [2]. Genetic traits influence the higher proanthocyanidin content in cranberry fruit samples [36]. Wang et al. indicated that a similar (high/low) proanthocyanidin content in fruit samples of related cranberry cultivars could be attributed to genetic factors influencing the synthesis of flavonoids [37].

Carpenter et al. carried out proanthocyanidin assays on fruit samples of the US-grown cranberry cultivars ‘Ben Lear’, ‘Bugle’, ‘Early Black’, ‘Howes’ and ‘Mullica Queen’. The authors indicated that the proanthocyanidin content of the fruit samples of these cultivars follows a decreasing trend, as follows: ‘Howes’ > ‘Mullica Queen’ and ‘Early Black’ > ‘Bugle’ > ‘Ben Lear’ [38]. The proanthocyanidin content of the fruit samples of ‘Ben Lear’, ‘Early Black’ and ‘Howes’ cultivars analyzed in our study followed a similar decreasing trend, as follows: ‘Howes’ > ‘Early Black’ > ‘Ben Lear’.

Lu et al. performed a quantitative analysis of proanthocyanidins in 21 fruit samples of *Vaccinium macrocarpon* cultivars grown in New Zealand and found that fruit samples of the cranberry cultivars ‘Crowley’ and ‘Howes’ have the highest levels of proanthocyanidins, suggesting that they are potential food resources for health use [32]. In our study, the highest levels of proanthocyanidins were found in fruit samples of the cranberry cultivar ‘Howes’, whereas the lowest levels of proanthocyanidins were found in fruit samples of the ‘Crowley’ cultivar. Climatic and geographical factors in different continents may differently influence the biosynthesis of proanthocyanidins in the fruit of the ‘Crowley’ and ‘Howes’ cultivars [39].

Cranberry fruit plant material is used in juice as well as in tablet and capsule formulations for the treatment and prevention of urinary tract infections, with proanthocyanidins mediating this effect [40]. Studies on the quantitative composition of proanthocyanidins allow for the identification of cranberry cultivars that accumulate the highest levels of proanthocyanidins in berries when grown under the climatic conditions of the Baltic region. Due to the high levels of proanthocyanidins detected in the fruit samples, the cranberry cultivars ‘Kalnciema Agrā’, ‘Kalnciema Tumšā’ and ‘Kalnciema Ražīgā’ developed in Latvia and the cranberry cultivars ‘Searles’ and ‘Howes’ developed in the USA are promising for introduction and cultivation in the climatic conditions in the Baltic region.

### 2.3. Determination of the Quantitative Composition of Flavanols in Fruit Samples of Large Cranberry Cultivars

Studies on the biological effects of flavonols detected in cranberry fruit samples have shown their anticancer [41,42], anti-inflammatory [43] and cardiovascular-system-improving [44] effects.

**Figure 5:**
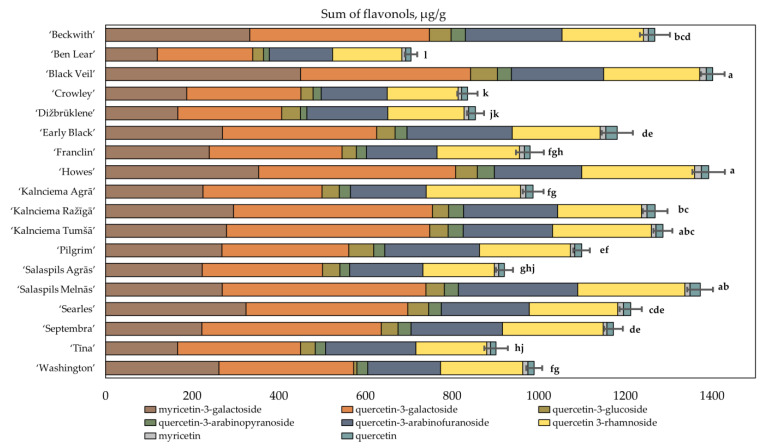
Variability in the total amount of flavonols in fruit samples of large cranberry cultivars. Statistically significant differences in total flavonols content between samples of cranberry cultivars are marked with different letters (*p* < 0.05).

We performed an analysis of the composition of flavonol compounds in cranberry fruit samples (Figure 5). We identified four major flavonols: myricetin-3-galactoside (16.89–32.14%), quercetin-3-galactoside (26.69–36.42%), quercetin-3-arabinofuranoside (14.45–23.13%) and quercetin-3-rhamnoside (14.85–22.70%), which were the predominant flavonols in the quantitative composition in the cranberry fruit samples. The amounts of the other compounds of the flavonol group were lower, including quercetin-3-glucoside (0.81–5.23%), quercetin-3-arabinopyranoside (1.75–2.80%), myricetin (0.74–1.23%) and quercetin (1.09–2.23%).

Wang et al. investigated the qualitative composition of flavonols in the fruit of the cranberry cultivars ‘Crimson Queen’, ‘Stevens’, ‘Ben Lear’, ‘Howes’, ‘Early Black’, ‘Demoranville’ and ‘Mullica Queen’ and found that myricetin-3-galactoside comprises 19–32%, quercetin-3-galactoside comprises 31–46%, quercetin-3-rhamnopyranoside comprises 7–14% and quercetin-3-arabinofuranoside comprises 7–17% of the total flavonol content [37]. These results are consistent with those of our study. Urbstaite et al. investigated the cranberry cultivars ‘Bawfay’, ‘Bergman’, ‘Profilic’, ‘Woolman’ and ‘Searles’ as well as the genetic clones ‘BL-12’ and ‘Bain-MC’ and determined the qualitative and quantitative compositions of flavonols. They found that quercetin-3-galactoside comprises 28.93–32.68%, myricetin-3-galactoside comprises 23.83–30.37%, quercetin-3-arabinofuranoside comprises 13.35–19.74% and quercetin-3-rhamnoside comprises 11.55–16.09% of the total amount of flavonols in cranberry fruit samples, which is in line with the quantitative composition of the flavonols found in the fruit samples of our studied cultivars [45].

We found that the flavonol content in cranberries varied from 705.15 ± 14.5 µg/g to 1401.79 ± 27 µg/g. The average total flavonol content of cranberry fruit samples was 1100.94 ± 26 µg/g. The highest total flavonol content (1401.79 ± 27 µg/g) was determined in cranberries of the ‘Black Veil’ cultivar, which was not statistically different from that found in the fruit samples of the cranberry cultivars ‘Howes’, ‘Kalnciema Tumšā’ and ‘Salaspils Melnās’ (Figure 5). The lowest total anthocyanin content (705.15 ± 14.5 µg/g) was found in cranberries of the ‘Ben Lear’ cultivar.

Gudžinskaitė et al. investigated the fruit samples of cranberry cultivars grown in a Lithuanian climate and established that the total flavonol content varied from 149.59 mg/g to 370.38 mg/g [46]. Oszmiański et al. analyzed the qualitative and quantitative composition of flavonols in fruit of cranberry cultivars grown under climatic conditions in Poland. They found that the total flavonol content in the fruit samples of the studied cultivars varied from 6430 µg/g to 10,880 µg/g [13]. In our study, cranberry fruit samples were found to have a lower total flavonol content compared to the levels found by Gudžinskaitė et al. and Oszmiański et al.

**Figure 6:**
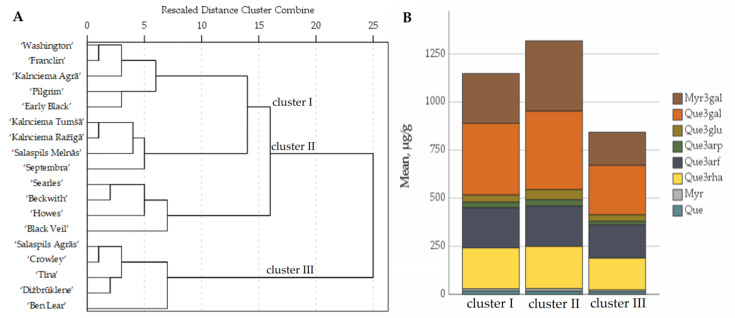
Dendrogram of distribution of cranberry cultivars into three groups according to flavonol content of the fruit samples (**A**). Graph of the distribution of the average flavonol content in groups (**B**). Myr3gal—myricetin-3-galactoside; Que3gal—quercetin-3-galactoside; Que3glu—quercetin-3-glucoside; Que3arp—quercetin-3-arabinopyranoside; Que3arf—quercetin-3-arabinofuranoside; Que3rha—quercetin-3-rhamnoside; Myr—myricetin; Que—quercetin.

The comparative analysis of the flavonol content of different large cranberry cultivars was conducted by applying hierarchical cluster analysis. The cluster analysis of the cultivar samples was carried out by investigating the results of studies on the qualitative and quantitative composition of flavonols in the fruit samples of different cultivars. Based on the quantitative compositions of flavonols in the cranberry fruit samples, the samples were divided into three clusters (Figure 6).

Cluster I consisted of fruit samples of the ‘Early Black’, ‘Franclin’, ‘Kalnciema Agrā’, ‘Kalnciema Ražīgā’, ‘Kalnciema Tumšā’, ‘Pilgrim’, ‘Salaspils Melnās’, ‘Septembra’ and ‘Washington’ cultivars, in which quercetin-3-galactoside was found to account for 32.18% ± 3.38% of the total flavonol content, and myricetin-3-galactoside was found to account for 22.72% ± 2.22% of the total flavonol content. The average total flavonol content of cluster I cranberry fruit samples was 1149 ± 26 µg/g.

Cluster II included the cranberry cultivars ‘Searles’, ‘Howes’, ‘Black Veil’ and ‘Beckwith’. Cluster II cranberry fruit samples were found to contain 1.5 times higher amounts of myricetin-3-galactoside than the average total myricetin-3-galactoside content of the tested cultivars, whereas the total flavonol content was higher by 1.2 times. In the samples of the cultivars belonging to the cluster, the quercetin-3-galactoside content was found to account for 31.50% ± 2.23% of the total amount of flavonols, and the myricetin-3-galactoside content was found to account for 27.67% ± 3.06% of the total amount of flavonols. Urbstaite et al. and Gudžinskaitė et al. performed comparative studies on the flavonol content of different cultivars and found that cranberries of the ‘Searles’ cultivar have the highest total flavonol content [45,46].

Cluster III included the cultivars ‘Ben Lear’, ‘Crowley’, ‘Salaspils Agrās’, ‘Tīna’ and ‘Dižbrūklene’, in which the total flavonol levels were 1.3 times lower than the average total flavonol levels of the tested cultivars. In the fruit samples of cluster III cultivars, quercetin-3-galactoside accounted for 29.87% ± 1.50% of the total flavonol content, and myricetin-3-galactoside accounted for 20.19% ± 2.60% of the total flavonol content.

Differences in the qualitative and quantitative compositions of phenolic compounds in the fruit samples of different cranberry cultivars may be influenced by genetic factors due to differences in the synthesis of secondary metabolites [47]. Myricetin-3-galactoside and quercetin-3-galactoside are the main components of bioactive compounds in cranberries, with different compositions in samples from cultivars of different clusters. Prasain et al. reported that myricetin exerts anticancer properties by inhibiting the progression of malignant prostate cancer, inducing apoptosis in colon cancer cells and inhibiting the growth of bladder cancer cells [48]. Quercetin found in cranberry fruit has effects on bladder [49], breast [50] and ovarian [51] cancer cells. Cranberry plant material with known qualitative and quantitative compositions of flavonols can be used to produce high-quality foods and food supplements, and the cranberry cultivars ‘Howes’, ‘Black Veil’ and ‘Salaspils Melnās’ with the highest flavonol content in fruit samples would be suitable for the production of such preparations.

### 2.4. Determination of the Quantitative Composition of Triterpenoids in Fruit Samples of Large Cranberry Cultivars

**Figure 7:**
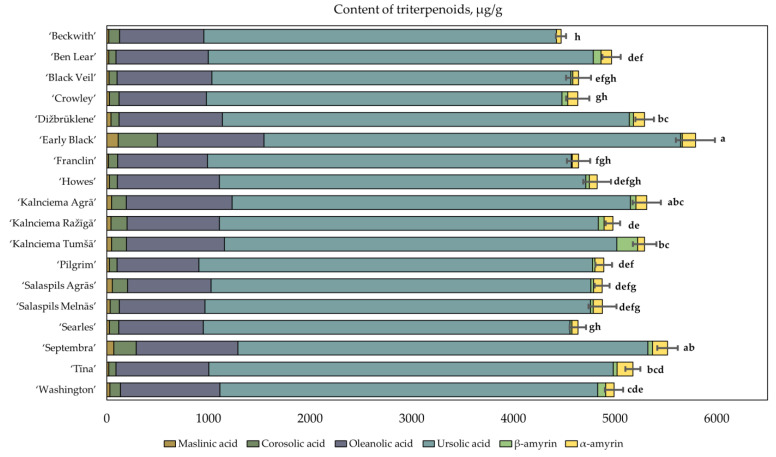
Variability in the total amount of triterpenoids in fruit samples of large cranberry cultivars. Statistically significant differences in total triterpenoid content between samples of cranberry cultivars are marked with different letters (*p* < 0.05).

Triterpenoids are a group of biologically active compounds. Triterpene compounds have been demonstrated to have antioxidant [52], anti-inflammatory [53] and anti-cancer effects [54,55]. In this study, we investigated the qualitative and quantitative compositions of triterpenoids in the fruit samples of cranberry cultivars grown under Latvian climatic conditions (Figure 7).

Our study showed that ursolic acid accounted for 70.79% to 79.20% of the total triterpenoid content in the fruit samples of the tested cranberry cultivars. Klavins et al. and Oszmiański et al. confirmed that ursolic acid is the predominant triterpene compound in cranberries [56,57].

The other compounds of the terpenoid group accounted for 20.80% to 29.21% of the total triterpenoid content. Of these, oleanolic acid accounted for 16.46% to 20.84%, corosolic acid accounted for 1.40% to 6.67%, maslinic acid accounted for 0.36% to 1.93%, α-amyrin accounted for 1.01% to 3.00%, and β-amyrin accounted for up to 1.93%. Šedbarė et al. studied samples of cranberry cultivars grown under Latvian climate and established that ursolic acid accounts for 68.31 ± 0.95%, oleanolic acid accounts for 15.75 ± 0.95%, corosolic acid accounts for 1.21 ± 0.37%, maslinic acid accounts for 0.17 ± 0.13%, and α-amyrin accounts for 0.52 ± 0.30% of the total triterpenoid content [34]. The results of the analyses are consistent with the triterpenoid composition established in the samples of the studied cultivars grown in the collection of the National Botanic Garden of Latvia.

The study of the triterpenoid composition showed that the total triterpenoid content of the cranberry fruits varied from 4468.77 ± 50 µg/g to 5791.49 ± 192 µg/g (Figure 7). The average total triterpenoid content of the cranberry fruit samples was 4988.72 ± 104 µg/g. The highest total triterpenoid content (5791.49 ± 192 µg/g) was established in cranberries of the ‘Early Black’ cultivar. Moreover, the lowest total triterpenoid content (4468.77 ± 50 µg/g) was established in cranberries of the ‘Beckwith’ cultivar, which was not statistically different from the total triterpenoid content of the fruit samples of the ‘Black Veil’, ‘Crowley’, ‘Franclin’, ‘Howes’ or ‘Searles’ cranberry cultivars. The coefficient of variation of the total triterpenoid content in the cranberries was 7%. The amount of the predominant compound—ursolic acid—in cranberry fruit samples varied from 3468.03 ± 35 µg/g to 4100.04 ± 144 µg/g, with a coefficient of variation lower than that of the total triterpenoid content (about 5%).

Sedbare et al. found that the total triterpenoid content of cranberry fruit samples varies from 4215.78 ± 45 to 6232.74 ± 123 µg/g [34]. The triterpenoid content determined by the authors is consistent with the results of our study. Oszmiański et al. found that the average composition of triterpenoids in the cranberry cultivars ‘Pilgrim’, ‘Stevens’ and ‘Ben Lear’ harvested at different maturity stages ranges from 2528.0 µg/g to 3201.5 µg/g [57]. In our study, the total amount of triterpenoids established in cranberries was about two-fold higher compared to the amount found in fruit samples by Oszmiański et al. [57]. Xue et al. performed triterpenoid analysis on fruit samples of the cranberry cultivars ‘Scarlet Knight’ and ‘Stevens’ and established that the triterpenoid levels vary from 36,720 mg/g to 144,370 mg/g, which is higher than that detected in our study [43].

**Figure 8:**
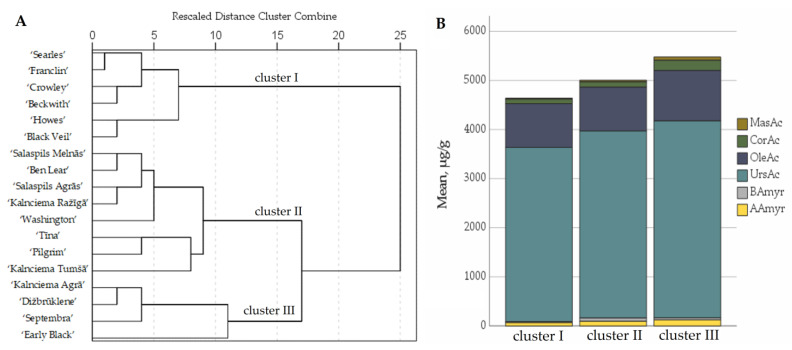
Dendrogram of distribution of cranberry cultivars into three groups according to triterpenoid content of the fruit samples (**A**). Graph of the distribution of the average triterpenoid content in the groups (**B**). MasAc—maslinic acid; CorAc—corosolic acid; OleAc—oleanolic acid; UrsAc—ursolic acid; BAmyr—β-amyrin; AAmyr—α-amyrin.

The comparative analysis of the quantitative composition of triterpenoids in cranberry cultivars was carried out by applying hierarchical cluster analysis, based on the quantitative composition of triterpenoids found in the fruit samples of different cultivars. The cranberry fruit samples were divided into three clusters based on their triterpenoid compositions (Figure 8).

Cluster I included the fruit samples of the cranberry cultivars ‘Searles’, ‘Howes’, ‘Franclin’, ‘Crowley’, ‘Black Veil’ and ‘Beckwith’, which were found to have lower levels of triterpenoids than the average total amount of triterpenoids in fruit samples of the tested cultivars. Cluster I included samples with low levels of maslinic acid and β-amyrin (respectively, 0.49% and 1.43%).

Cluster II included the fruit samples of the cranberry cultivars ‘Ben Lear’, ‘Tīna’, ‘Salaspils Agrās’, ‘Salaspils Melnās’, ‘Pilgrim’, ‘Kalnciema Tumšā’, ‘Kalnciema Ražīgā’ and ‘Washington’, in which the total triterpenoid content was found to correspond to the total average triterpenoid content in the fruit samples of the tested cultivars.

Cluster III included the fruit samples of the cranberry cultivars ‘Kalnciema Agrā’, ‘Septembra’, ‘Dižbrūklene’ and ‘Early Black’, in which the total triterpenoid content was higher than the average total triterpenoid content in the fruit samples of the tested cultivars. The chemical compositions of the triterpenoids in the fruit samples of cluster III cranberry cultivars ‘Septembra’ and ‘Early Black’ were characterized by the highest levels of maslinic acid and corosolic acid (respectively, 1.20% and 3.74%).

Qualitative and quantitative analysis of the triterpenoid compositions of cranberry fruit samples showed that ursolic acid, together with oleanolic acid, comprised 94% of the total triterpenoid content. Ursolic and oleanolic acids detected in cranberries have antioxidant [58], anti-inflammatory [59] and anticancer [60] effects, making them relevant components in foods or dietary supplements for disease prevention. Cranberry plant material is a natural source of preparation whose effects are mediated by triterpene compounds, in particular, ursolic acid and oleanolic acid. Fruit samples of the cranberry cultivar ‘Early Black’ had the highest levels of ursolic and oleanolic acids, and the raw material of cranberry fruit of this cultivar can therefore be used as a natural source of these triterpenic acids.

## 3. Materials and Methods

### 3.1. Reagents

All solvents, reagents, and standards were of analytical reagent grade. Acetone, methanol, acetonitrile, hydrochloric acid, 4-(Dimethylamino) cinnamaldehyde and reference standarts for β-Amyrin, maslinic acid, oleanolic acid, corosolic acid, ursolic acid, α-Amyrin, quercetin-3-rhamnoside, myricetin, quercetin-3-α-L-arabinopyranoside and quercetin-3-α-L-arabinofuranoside were purchased from Sigma-Aldrich (Steinheim, Germany). Ethanol 96% (*v*/*v*) was purchased from AB Stumbras (Kaunas, Lithuania), formic acid was bought from Merck (Darmstadt, Germany), and Trolox (6-hydroxy-2,5,7,8-tetramethylchroman-2-carboxylic acid) was purchased from Scharlau (Sentmenat, Spain). Reference standards for myricetin-3-galactoside, cyanidin chloride, peonidin chloride, malvidin-3-galactoside, malvidin-3-arabinoside, malvidin chloride, peonidin-3-arabinoside, cyaniding-3-galactoside, delphinidin-3-galactoside, peonidin-3-glucoside, peonidin-3-galactoside, cyaniding-3-glucoside and cyaniding-3-arabinoside were purchased from Extrasynthese (Genay, France). Standards for quercetin and quercetin-3-galactoside were purchased from Carl Roth (Karlsruhe, Germany). The standard for quercetin-3-O-glucoside was bought from Biochemistry (Buchs, Switzerland). Purified deionized water was provided using a water purification system (Milli-Q^®^, Millipore, Bedford, MA, USA).

### 3.2. Plant Material

The object of the research was samples of different cultivars of large cranberries (*Vaccinium macrocarpon* Aiton) grown in the climatic conditions of Latvia, in the collection of the National Botanic Garden (Salaspils, Latvia) (56°51′55.4″ N 24°21′36.9″ E). The climate in Latvia is temperate and transitional from maritime to continental [61]. The average yearly temperature is 6.8 °C [62]. The following cranberry cultivars were collected on October 14, 2021: ‘Beckwith’, ‘Ben Lear’, ‘Black Veil’, ‘Crowley’, ‘Franclin’, ‘Kalnciema Agrā’, ‘Kalnciema Tumšā’, ‘Pilgrim’, ‘Salaspils Agrās’, ‘Salaspils Melnās’, ‘Searles’, ‘Septembra’ and ‘Tīna’. The cranberry cultivars ‘Dižbrūklene’, ‘Early Black’, ‘Howes’, ‘Kalnciema Ražīgā’ and ‘Washington’ were collected on October 18, 2021. Cranberry fruit samples were stored in a freezer at −20 °C and then transferred to an ultra-low-temperature freezer at −60 °C (CVF330/86, ClimasLab SL, Barcelona, Spain). In the next step, cranberries were lyophilized using the methodology described by Gudžinskaitė et al. [46]. Freeze-dried cranberries were powdered in an electric mill (Retsch GM 200, Retsch GmbH, Hahn, Germany). Loss on drying was estimated according to the method specified in the European Pharmacopoeia [63].

### 3.3. Extraction Procedure

Extraction procedures of anthocyanins, proanthocyanidins and flavonols from cranberry fruit samples were performed using the methodologies described by Urbstaite et al. [23]. During the analysis, 1 g of lyophilizate cranberry powder (exact weight) was weighed and extracted with 70% (*v*/*v*) ethanol containing 1% hydrochloric acid in an ultrasonic bath (Elmasonic P, Elma Schmidbauer GmbH, Singen, Germany) for 15 min at 80 kHz and 565 W at room temperature. Each lyophilized cranberry sample was extracted three times. The extracts were filtered into a 20 mL volumetric flask.

Extraction procedures of triterpenoids from cranberry fruit samples were performed using the methodologies described by Sedbare et al. [64]. During the analysis, 1 g of lyophilizate cranberry powder (exact weight) was weighed and extracted with 10 mL of 100% *(v/v)* acetone in an ultrasonic bath (Elmasonic P, Elma Schmidbauer GmbH, Singen, Germany) for 60 min from 22 ± 1 °C to 60 ± 1 °C at 1130 W and 80 kHz. Each lyophilized cranberry sample was extracted three times. The extracts were filtered into a 10 mL volumetric flask.

The produced extracts were stored in dark glass containers at −20 °C. Extracts of cranberry samples were filtered through membrane filters (pore size 0.22 µm, Carl Roth GmbH, Karlsruhe, Germany) prior to chromatographic analysis.

### 3.4. Chromatographic Methods

The qualitative and quantitative compositions of anthocyanins, phenolic compounds and triterpenoids in cranberry fruit were determined using an Ultra-High-Performance Liquid Chromatography system (Waters ACQUITY UPLC, Milford, MA, USA) with a photodiode array detector (ACQUITY UPLC PDA eλ, Milford, MA, USA). Compounds were separated using an ACE C18 reverse phase (100 × 2.1 mm, 1.7 µm particles) column (An Avantor ACE, ACT, Aberdeen, UK). During analysis, 20% acetonitrile (*v*/*v*) solvents were used for an autosampler needle-washing step.

The amounts of identified compounds were estimated from the calibration curves and expressed as µg/g dry weight. Information about the parameters (LOD, LOQ, linearity range, calibration curve and R^2^) of all the identified compounds used in the chromatography methods is described in the Supplementary Materials in Table S1.

#### 3.4.1. Determination of Anthocyanins and Anthocyanidins

The content of anthocyanins and anthocyanidins in cranberry fruit samples was determined according to the methodology validated by Vilkickyte et al. [65]. The volume of cranberry extract used for analysis was 1 μL, and the flow rate was 0.5 mL/min. The mobile phase was a gradient prepared by mixing 100% acetonitrile (*v*/*v*) (solvent A) and aqueous 10% formic acid solution (*v*/*v*) (solvent B), as follows: 0.0–2.0 min, 5% B; 2.0–7.0 min, 9% B; 7.0–9.0 min, 12% B; 9.0–10.0 min, 25% B; 10.0–10.5 min, 80% B; 10.5–11.0 min, 80% B; and 11.0–12.0 min, 5% B. The column temperature was maintained at 30 °C. All the identified anthocyanins and anthocyanidins were quantified at a wavelength of 520 nm. Chromatograms of cranberry fruit samples and the standards mixture are provided in the Supplementary Materials (Figure S1).

#### 3.4.2. Determination of Flavonols

The content of flavonols in cranberry fruit samples was determined according to the methodology developed and validated by Urbstaite et al. [45]. The volume of cranberry extract used for analysis was 1 μL, and the flow rate was 0.5 mL/min. The mobile phase was a gradient prepared by mixing 100% acetonitrile (*v*/*v*) (solvent A) and aqueous 0.1% formic acid solution (*v*/*v*) (solvent B), as follows: 0 min, 5% B; 1 min, 12% B; 3 min, 12% B; 4 min, 13% B; 9 min, 25% B; 10.5 min, 30% B; 12 min, 30% B; 12.5 min, 90% B; 13 min, 90% B; 13.5 min, 5% B; and 14.5 min, 5% B, delaying the next injection by 2 min. The column temperature was maintained at 30 °C. All the identified flavonols were quantified at a wavelength of 360 nm. Chromatograms of cranberry fruit samples and the standards mixture are provided in the Supplementary Materials (Figure S2).

#### 3.4.3. Determination of Triterpenoids

The content of triterpenoids in the cranberry fruit samples was determined according to the methodology developed and validated by Sedbare et al. [64]. The volume of cranberry extract used for analysis was 1 μL, and the flow rate was 0.2 mL/min. The mobile phase was a gradient prepared by mixing 0.1% formic acid (*v*/*v*) (solvent A) and 100% methanol (*v*/*v*) (solvent B), as follows: 0 min, 92% B; 8 min, 97% B; 9 min, 98% B; 29.5 min, 98% B; and 30 min, 92% B, delaying the subsequent injection by 10 min. The column temperature was maintained at 25 °C. All the identified triterpenoids were quantified at a wavelength of 205 nm. Chromatograms of cranberry fruit samples and the standards mixture are provided in the Supplementary Materials (Figure S3).

### 3.5. Spectrophotometric Studies

The total proanthocyanidin content was estimated using the DMCA (4-(Dimethylamino)cinnamaldehyde) method [66]. An amount of 10 μL of cranberry extract was mixed with 3 mL DMCA (0.1% DMCA (*m*/*v*) in ethanol-hydrochloric acid: 9:1 (*v*/*v*)), and the absorbance of this solution was measured at 640 nm after 5 min on a spectrophotometer (M550 UV/Vis, Spectronic CamSpec, Garforth, UK). The DMCA solution in acidified ethanol served as the reference solution. The total proanthocyanidin content was estimated from the (–)-epicatechin (0.0625–1 mg/mL) calibration curve (𝑦 = 0.7021𝑥 + 0.0138; R2 = 0.9994) and expressed as µg/g (–)-epicatechin equivalent (EE) dry weight.

### 3.6. Data Analysis

Data analysis was performed using the computer software programs Microsoft Excel 2016 (Microsoft, Rednmond, WA, USA) and SPSS Statistics 21 (IBM, New York, NY, USA). During the study, each cranberry extract was evaluated using one-time chromatographic and spectrophotometric tests. The averages and standard deviations (SD) of the independent evaluations of three technical replicates were calculated. The Kruskal–Wallis A-way ANOVA test for multiple comparisons was used to evaluate differences in the amounts of identified compounds between fruit samples of cranberry cultivars. Hierarchical cluster analysis, applying the complete-linkage clustering method with Euclidean distances, was performed to determine the groupings of cranberries. The level of significance was chosen as *p* < 0.05.

## 4. Conclusions

A comparative analysis of the qualitative and quantitative compositions of anthocyanins, proanthocyanidins, flavonols and triterpenoids in the fruit samples of cranberry cultivars showed that the phytochemical compositions of the fruit samples of the studied cultivars varied and that different cultivars were able to accumulate different amounts of biologically active compounds in their fruit. This study did not evaluate the influence of environmental factors and fruit development on the amount of biologically active compounds in cranberry fruit samples. However, a detailed investigation of the phytochemical composition of introduced and bred large cranberry fruits from the collection of the National Botanic Garden of Latvia was carried out for the first time. The results of our research show that the chemical compositions of cranberries of the cultivars bred in Latvia correspond to the phytochemical compositions of cranberries of the cultivars bred in the United States. Thus, the results of the chemical compositions of the cranberries indicate that the cultivars bred in Latvia are quite promising for introduction and cultivation in the Baltic countries.

The analysis of the quantitative composition of proanthocyanidins showed that the highest levels of these compounds were present in fruits of the cranberry cultivars ‘Kalnciema Agrā’, ‘Kalnciema Tumšā’ and ‘Kalnciema Ražīgā’ bred in Latvia, and in the fruits of the cranberry cultivars ‘Searles’ and ‘Howes’ bred in the United States. The highest levels of flavonols were found in the cranberries of cultivars ‘Kalnciema Tumšā’, ‘Salaspils Melnās’, ‘Howes’ and ‘Black Veil’. The highest levels of anthocyanins were found in cranberries of the ‘Franclin’ cultivar. The analysis of the quantitative compositions of triterpene compounds showed that the cranberries of the ‘Early Black’ cultivar contained the highest levels of triterpene compounds. The cranberry fruit is a natural plant source of anthocyanins, proanthocyanidins, flavonols and triterpenoids, which are groups of biologically active compounds, and it is therefore promising to carry out the breeding, introduction and cultivation of cranberry cultivars in order to produce new high-quality cranberry fruit preparations with antioxidant and other health benefit effects.

## Data Availability

All data generated during this study are included in this article.

## Supplementary Materials

The following supporting information can be downloaded at: https://www.mdpi.com/article/10.3390/plants12040771/s1, Table S1: Parameters of the identified compounds; Figure S1: UHPLC-PDA chromatograms (λ = 520 nm) of the anthocyanin and anthocyanidin standards mixtures and large cranberry extracts of cultivars ‘Franclin’ and ‘Washington’; Figure S2: UHPLC-PDA chromatograms (λ = 360 nm) of flavonol standards mixture and the large cranberry extracts of cultivars ‘Ben Lear’ and ‘Black Veil’; Figure S3: UHPLC-PDA chromatograms (λ = 205.5 nm) of the triterpenoid standards mixture and the large cranberry extracts of cultivars ‘Early Black’ and ‘Beckwith’.
