# Supplementary material for: Phytochemical Composition of the Fruit of Large Cranberry (Vaccinium macrocarpon Aiton) Cultivars Grown in the Collection of the National Botanic Garden of Latvia
Source: Plants (Basel). 2023 Feb 8;12(4):771. doi: 10.3390/plants12040771 (PMC9964011; doi:10.3390/plants12040771)
Supplement: Supplementary file 1 [file plants-12-00771-s001.zip › plants-2177195-supplementary.pdf]

Supplementary material:

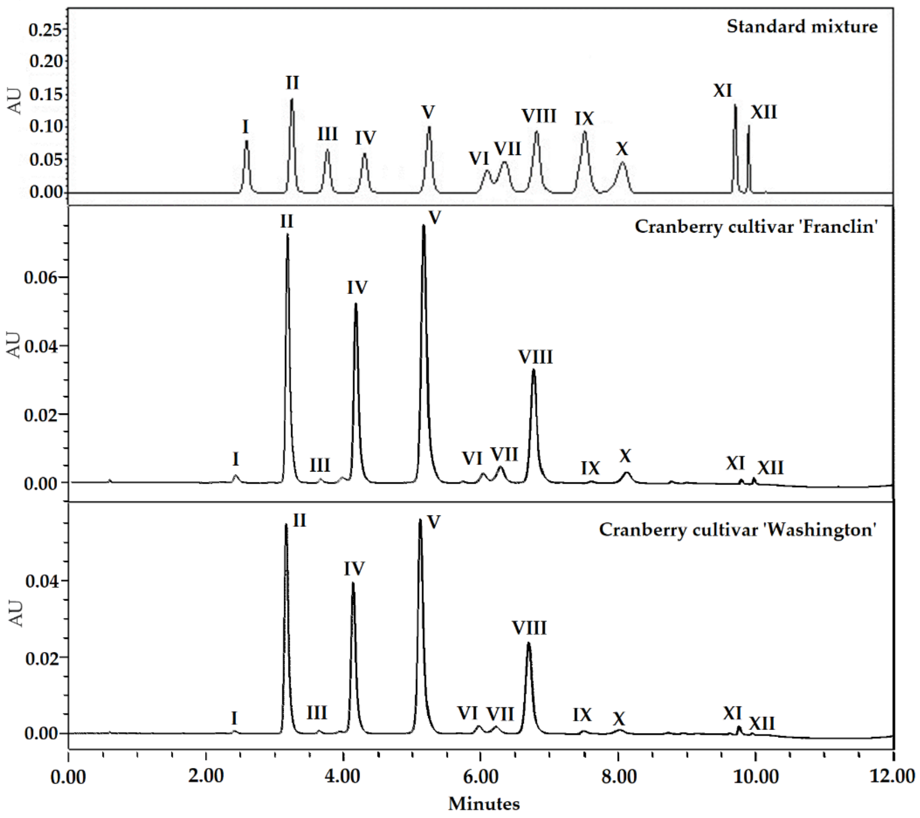

Figure S1: UHPLC-PDA chromatograms (λ = 520 nm) of the anthocyanin and anthocyanidin standards mixtures and large cranberry extracts of cultivars 'Franclin' and 'Washington'. The compounds of the identified peaks are described in Table S1.

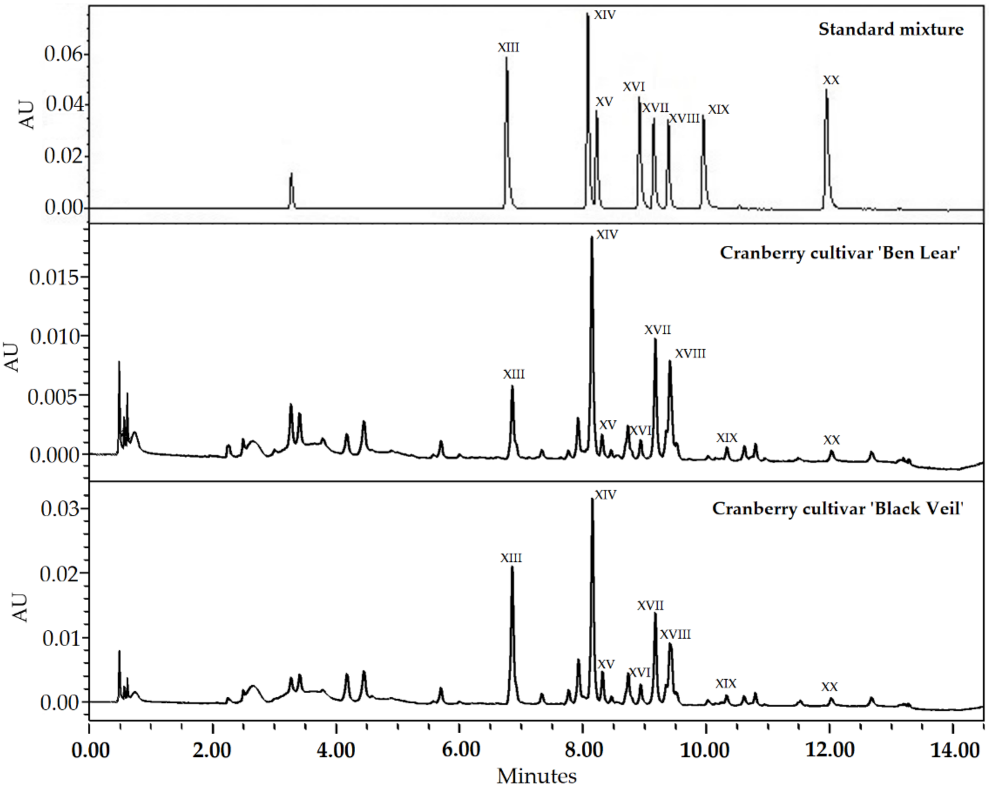

Figure S2: UHPLC-PDA chromatograms (λ = 360 nm) of flavonol standards mixture and the large cranberry extracts of cultivars 'Ben Lear' and 'Black Veil'. The compounds of the identified peaks are described in Table S1.

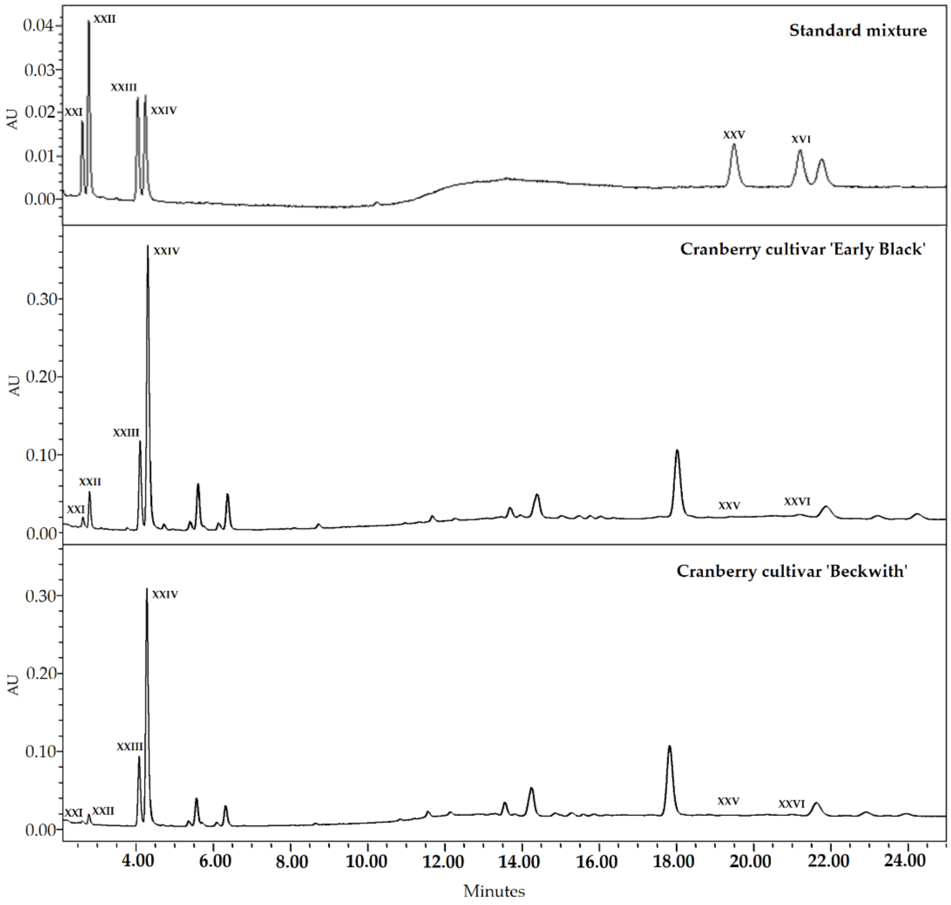

Figure S3: UHPLC-PDA chromatograms (λ = 205.5 nm) of the triterpenoid standards mixture and the large cranberry extracts of cultivars 'Early Black' and 'Beckwith'. The compounds of the identified peaks are described in Table S1.

Table S1. Parameters of the identified compounds

Peak	Compound	Linearity range (μg/mL)	Calibration equation	R^2	LOD	LOQ
I	Delphinidin-3-galactoside	3.91–125.00	$y = 4900\ x - 3480$	0.999	1.15	3.19
II	Cyanidin-3-galactoside	0.78–125.00	$y = 4940\ x - 2370$	0.999	0.16	0.53
III	Cyanidin-3-glucoside	3.13–100.00	$y = 4230\ x + 1610$	0.999	0.10	3.07
IV	Cyanidin-3-arabinoside	3.13–100.00	$y = 4800\ x + 2220$	0.999	0.93	3.11
V	Peonidin-3-galactoside	3.125–100	$y = 5320\ x + 8770$	0.999	0.35	2.53
VI	Peonidin-3-glucoside	0.98–125.00	$y = 3970\ x - 1430$	0.999	0.30	0.99
VII	Malvidin-3-galactoside	3.13–100.00	$y = 6890\ x + 3110$	0.999	1.00	3.09
VIII	Peonidin-3-arabinoside	3.125–100	$y = 5940\ x + 5320$	0.999	0.42	1.36
IX	Cyanidin	0.78–100.00	$y = 10400\ x - 1930$	0.999	0.07	0.24
X	Malvidin-3-arabinoside	0.78–125.00	$y = 5950\ x + 1590$	0.999	0.35	1.10
XI	Peonidin	1.56–100.00	$y = 7010\ x +1020$	0.999	0.33	1.09
XII	Malvidin	3.13–100.00	$y=1150\ x + 171$	0.999	0.59	1.96
XIII	Myricetin-3-galactoside	0.78–100	$y = 3450\ x - 396$	0.999	0.18	0.54
XIV	Quercetin-3-galactoside	3.13–200	$y = 4880\ x + 1180$	0.999	1.01	3.06
XV	Quercetin-3-glucoside	3.13–50	$y = 4160\ x - 61{,}7$	0.999	0.92	2.78
XVI	Quercetin-3-α-L-arabinopyranoside	3.13–50	$y = 5250\ x + 861$	0.999	0.70	2.12
XVII	Quercetin-3-α-L-arabinofuranoside	3.13–50	$y = 4170\ x - 199$	0.999	0.99	3.03
XVIII	Quercetin-3-rhamnoside	3.13–50	$y = 3690\ x + 797$	0.999	0.76	2.29
XIX	Myricetin	1.56–50	$y = 5360\ x - 1240$	0.999	0.45	1.36
XX	Quercetin	3.13–50	$y = 7450\ x - 1070$	0.999	0.76	2.29
XXI	Maslinic acid	3.125–200	$y = 2790\ x + 3990$	0.999	0.89	2.95
XXII	Corosolic acid	3.125–200	$y = 3280\ x + 750$	0.999	0.66	2.21
XXIII	Oleanolic acid	2.344–600	$y = 3240\ x + 12900$	0.999	0.55	1.85
XXIV	Ursolic acid	3.906–2000	$y = 2930\ x + 39000$	0.999	0.54	1.81
XXV	β-Amyrin	6.250–200	$y = 3170\ x + 6470$	0.999	1.11	3.69
XXVI	α-Amyrin	6.250–200	$y = 3090\ x - 1030$	0.999	1.37	4.58
